# Identification of the driving forces of climate change using the longest instrumental temperature record

**DOI:** 10.1038/srep46091

**Published:** 2017-04-07

**Authors:** Geli Wang, Peicai Yang, Xiuji Zhou

**Affiliations:** 1 LAGEO Institute of Atmospheric Physics, Chinese Academy of Sciences, Beijing, China; 2 Chinese Academy of Meteorological Sciences, Beijing, China

## Abstract

The identification of causal effects is a fundamental problem in climate change research. Here, a new perspective on climate change causality is presented using the central England temperature (CET) dataset, the longest instrumental temperature record, and a combination of slow feature analysis and wavelet analysis. The driving forces of climate change were investigated and the results showed two independent degrees of freedom —a 3.36-year cycle and a 22.6-year cycle, which seem to be connected to the El Niño–Southern Oscillation cycle and the Hale sunspot cycle, respectively. Moreover, these driving forces were modulated in amplitude by signals with millennial timescales.

Causality analysis in climate change is an active and challenging research area that remains highly uncertain. The Intergovernmental Panel on Climate Change (IPCC)^1^ advocates that human activity is the most important driving force of climate change, while some researchers have argued that natural forces might be the main cause. These different views are mainly due to a lack of methods to address the complexity of climate system and insufficiency in observational climate data.

Global circulation model (GCM) simulations are generally used to investigate the causality of climate change. However, due to the limited knowledge of the climate system, large uncertainties are still associated with GCMs; therefore, the improvement of current GCMs to meet the requirements for causality analysis is still an urgent issue. An alternative method to GCMs is to use long-term observational climate data to study the driving forces of climate change, a method that has recently benefited from the great progress made by physical and biological scientists in studying the driving forces in non-stationary time series. The main advantage of this approach is that observational data can be used to directly extract the driving forces of an unknown dynamical system. This can be achieved by two techniques. The first technique involves finding the driving forces by studying the connections among different physical factors. These types of relations cannot be established using general correlation analysis, but only in dynamical directional influences. Granger causality^2^ is a pioneering approach for achieving this task. Mutual information and transfer entropy^3^ are used to identify cause-effect relationships between components which is equivalent to Granger causality in the linear case and some attempts have been made to extend Granger causality to the nonlinear case^4^,^5^. Recently, Sugihara *et al*.^6^ presented another effective method known as convergent cross-mapping (CCM) to justify causality in some biological complex systems. Tsonis *et al*.^7^ used CCM to identify a causal relationship between cosmic rays and interannual variation in global temperature.

The second technique is to directly extract the driving force information behind the observational data. For example, cross-prediction error^8^ and slow feature analysis (SFA)^9^ have been successfully applied to extract slowly changing driving forces from non-stationary time series. To evaluate SFA, a modified logistic map has been used to test the ability of SFA to construct the driving forces from an observational time series, and the results showed that there is a good agreement between the constructed and the true driving forces with a correlation coefficient of 0.998^10^. This suggests that SFA is suitable for extracting the driving force from observational time series.

Using SFA and the wavelet transformation technique, Yang *et al*.^11^ (hereafter, Yang16) reconstructed and analyzed the driving forces for the monthly mean surface air temperature anomaly time series in the Northern Hemisphere, and found that the driving forces for this temperature climate system included two independent degrees of freedom that represented the effects of a 22-year solar cycle and the Atlantic Multidecadal Oscillation (AMO) on the climate system. Furthermore, they found that the driving forces are modulated in amplitude by signals with much longer time periods, this is, a long-term natural trend determined by the modulating amplitude signals.

The application of this method to climate change, which involves nonlinear and complex systems, is at a preliminary stage. The difficulties inherent in climate signal detection led us to further investigate the mechanism of the driving forces of the climate system. The present analysis for the temperature anomaly time series in the Northern Hemisphere needs to be verified and increased excavating and understanding of the causal effects directly from climatic observations is necessary with the longest instrumental record, the central England temperature (CET) dataset, which covers the Little Ice Age and some episodes of natural and anthropogenic warming of multidecadal duration.

## Results

**Figure 1:**
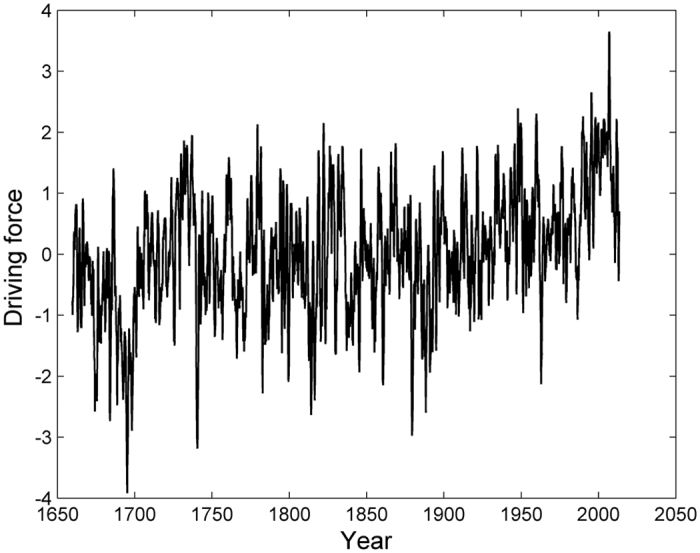
The Driving force constructed using CET dataset and SFA with embedding dimension m = 13. This figure was produced by using the MATLAB version R2010a software (http://cn.mathworks.com).

By using the SFA approach, we analyzed the reconstructed the driving force of climate change based on the CET dataset. Monthly mean surface air temperature data from January 1659 to December 2013 were used. Figure 1 shows the first output of the driving force constructed from the CET dataset, in which the embedding dimension was chosen as 13, which corresponds to a 13-month-window length (approximately one-year resolution)^11^ and the delay time *τ* was taken as 1.

**Figure 2:**
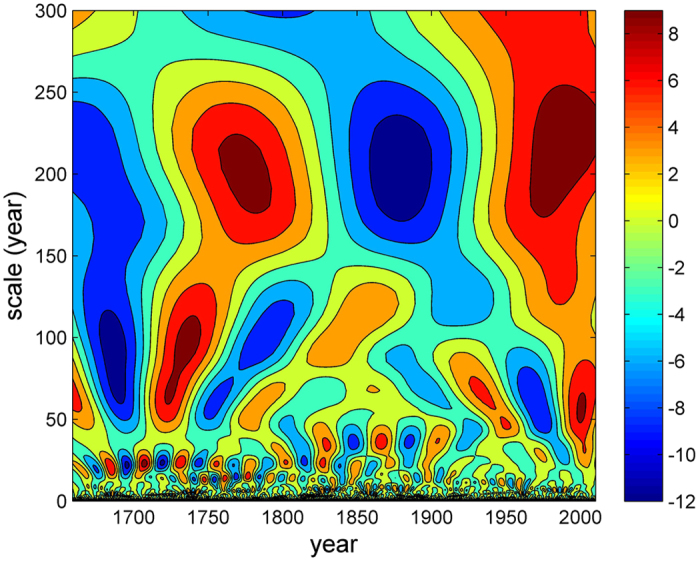
Real part of the wavelet transform coefficient for the driving force. This figure was produced using the MATLAB version R2010a software (http://cn.mathworks.com).

Next, by using the wavelet transformation technique^12^, we analyzed the scale structure of the constructed driving force and the corresponding physical meaning. Figure 2 shows the local wavelet power spectrum of the driving force output using the Morlet wavelet. As can be seen, there are some major spectral scales, which occur on decadal to centennial time scales.

**Figure 3:**
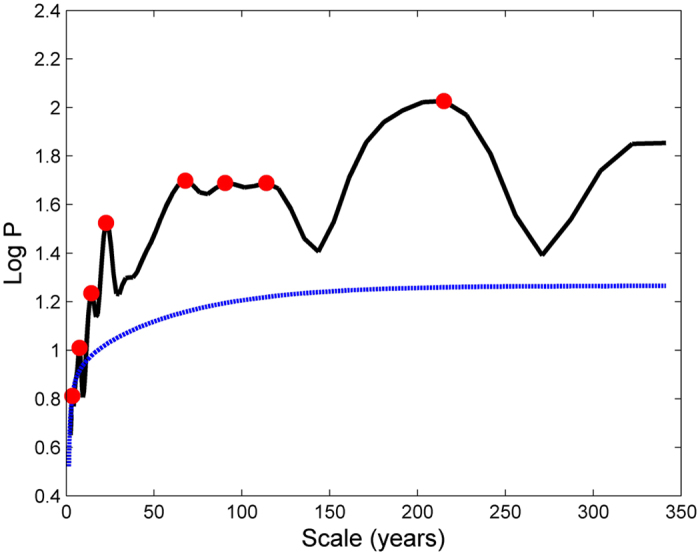
The time-averaged power spectrum of the driving force. This figure was produced by using the MATLAB version R2010a software (http://cn.mathworks.com).

To show the detailed characteristics of each spectral band based on the wavelet analysis of the driving force output, the time-averaged power spectrum of the driving forces is shown in Fig. 3. There are several spectral bands and the characteristic periods occur as spectrum peaks (red dots in Fig. 3) at 3.36, 7.5, 14.5, 22.6, 67.7, 90.4, 113.9 and 215 years, named L_1_ to L_8_, respectively. The blue dotted line in Fig. 3 is the 95% confidence level, and all scales pass the confidence test at the 95% level.

The peak L_1_ = 3.36 years seems to empirically correspond to the El Niño-Southern Oscillation (ENSO) signal, which has a period range of within 3 to 6 years. ENSO is arguably the most important global climate pattern and the dominant mode of climate variability^13^. The effect of ENSO on climate in Europe has been studied intensively using both models and observational or proxy data e.g. refs 14, 15, and a consistent and statistically significant ENSO signal on the European climate has been found e.g. refs 14 and 16.

The peak L_4_ = 22.6 years is coincident with the Hale sunspot cycle. This result partly agrees with some of the conclusions of Yang16. Solar variability has been shown to be a major driver of climate in central Europe during the past two millennia using Δ^14^C records. Furthermore, this result is essentially in good agreement with the findings of Scafetta e.g. refs 17, 18, 19, who found that the climate system was mostly characterized by a specific set of oscillations and these oscillations (61, 115, 130 and 983 years) appeared to be synchronous with major astronomical oscillations (solar system, solar activity and long solar/lunar tidal cycles).

The other peaks can be approximately expressed as linear combinations of L_1_ and L_4_: L_2_ = 7.5 ≈ 1/3L_4_; L_3_ = 14.5 ≈ L_4_−L_2_; L_5_ = 67.7 ≈ 3L_4_; L_6_ = 90.4 = 4L_4_ as the Gleisberg solar cycle; L_7_ = 113.9 ≈ 5L_4_; and L_8_ = 215 is a Suess solar cycle. From this, it is evident that these scales have harmonic relationships generated by the nonlinear interaction between L_1_ and L_4_^20^. Therefore, the results suggest that the oscillations found here are induced by ENSO and the Hale sunspot frequency, as all the peaks from the CET data are harmonics of the solar cycle and the ENSO cycle. For L_2_ = 7.5 years, oscillations with a period of about 7–8 years have been observed in some instrumental records in Europe, including the CET record^21^,^22^. Recently, by quantifying the cross-scale information transfer, it has been shown that the effect of this 7–8-year cycle can influence the interannual variability of air temperature anomalies^23^,^24^. This result is in contrast to the results of Yang16, in which the scale signal at 65.8 years was thought to be the AMO signal; however, in this study, a similar scale signal at 67.7 years is regarded as a harmonic of the solar cycle due to the approximately harmonic relationships.

**Figure 4:**
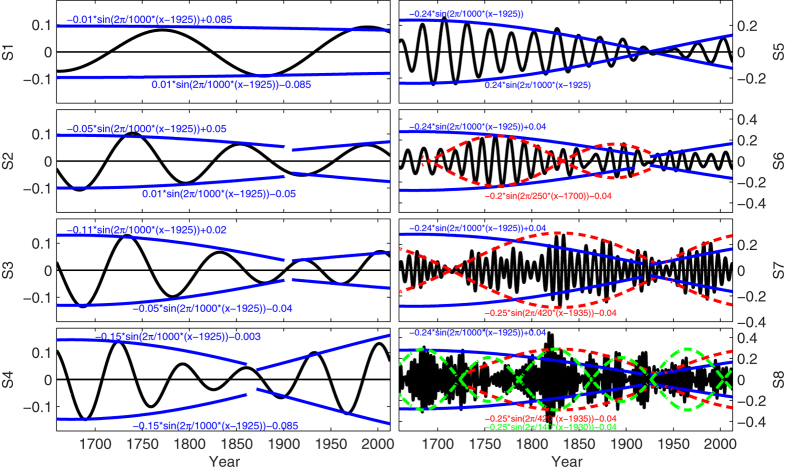
The band-pass filtering for S1-S8, where the black curves indicate the filter signals themselves, while the blue.red and green curves indicate their modulating signals from M1 to M8. This figure was produced by using the MATLAB version R2010a software (http://cn.mathworks.com).

To investigate each scale in detail, Fig. 4 shows the variation of the individual scale components (S_1_–S_8_ in Fig. 4) from band-pass filtering, which indicates the features of the driving forces in the time domain. Figure 4 indicates that all the signals are all strongly amplitude and phase modulated. The band-pass filters for the decomposed scale components, termed S_1_ to S_8_ from low to high frequency, were modulated in amplitude, suggesting that these signals are modulated by other signals with longer timescales. For fitting the envelopes of each filtered signal, the modulating signals, named M_1_ to M_8_, are presented using sine functions. The modulating signals can be approximately expressed as:


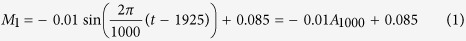



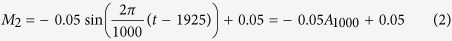



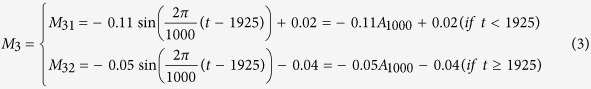



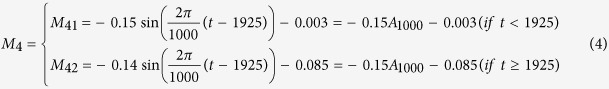



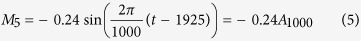



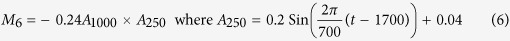



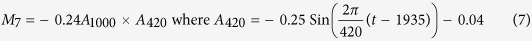



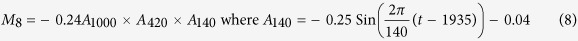


For convenience, 
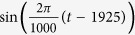
 is expressed as *A*_1000_. It is noted that all the modulating signals M_1_ to M_8_ have an oscillation period of about 1000 years and the amplitudes appear to increase with increasing frequency. The time phase of the zero amplitude of the modulating signal, specifically the year 1925 in *A*_1000_, is of particular interest, and is called the ‘zero phase point’. It denotes the point at which the driving forces change from decreasing to increasing. All the modulating amplitude signals that are connected to the millennial scale, suggest that the recent global warming process intensified from 1925 onwards. In particular, it is noted that modulations M_3_ and M_4_ have segmented structures, and modulations M_6_ and M_7_ have a hierarchical structure with a scale of 250 years in M_6_ and 420 years in M_7_. The modulating signal of S_8_ has a more complex structure, which contains multiple modes (420 years and 140 years) connected under *A*_1000_.

These modulating signals with periods of about 1000 years were also found for temperature in the Northern Hemisphere in Yang16, and control the amplitude changes of the driving forces and thus the input of energy. These modulating signals may play a key role in central England temperature and/or global warming. As such, an explanation for the physical background or origin of the amplitude changes should be provided. However, due to a lack of direct evidence, we are not yet able to clearly identify what they represent in physics using the techniques used in this study. More independent knowledge on climate change or a reasonable physical hypothesis is required. As for the most important long periodic signals of 1000 years, a reasonable speculation is that they represent the impacts of greenhouse gases (GHGs) on the climate system. This long period signal is topmost modulated which controls all scale-components, while GHGs is almost the unique factor to directly heat the air by absorbing the longwave radiation from the Earth surface. Thus, it should be considered that this millennial signal may be an impact of GHGs.

This result differs from the conclusions in Scafetta’s papers[e.g. refs 17, 18, 19], where this millennial period scale (983 years in his papers) is regarded as a harmonic of the solar cycle, but here we prefer to regard it as the GHG signal for the physical energy of the climate system.

The modulating signals in high-frequency scales have more complex structures due to the manifestation of new modulation modes with periods of 420, 250 or 140 years connected under *A*_1000_. They are superimposed on the modulating signals and jointly control climate change in central England for past 300 years and may be locally representative in spatial extent.

## Discussion and Conclusions

A new investigation on climate change causality is given using the longest instrumental temperature record — the CET dataset— which was analyzed using SFA and wavelet analysis. This investigation into the driving forces of climate change reproduces a 3.36-year cycle and a 22.6-year cycle, which may be connected to the ENSO cycle and the Hale sunspot cycle, respectively. Other beats from interdecadal to centennial components were also reproduced at 7.5, 14.5, 67.7, 90.4, 113.9 and 215 years, which could also be induced by ENSO and the Hale sunspot cycle as they are harmonics of the two basic frequencies. They are all strongly amplitude and phase modulated, and the modulating signals acting on the scale components are oscillations with a period of about 1000 years, which represent the impacts of GHGs as presented using the surface air temperature time series in the Northern Hemisphere in Yang16.

Tung and Zhou^25^ presented an interesting analysis result for the scale structure of the CET time series and found a scale component with a spectrum band from 50 to 90 years that propagates through the phase space of the indices considered as the AMO, due to the large thermal inertia associated with slow oceanic processes. This scale signal is reflected in the Northern Hemisphere area-averaged surface temperature signal and the CET dataset, which explains, by inference, a large fraction of the multidecadal non-uniformity of the observed global surface temperature warming in the twentieth century. However, the relationship between the 67.7 years found in this paper and the solar scales suggests that this climate component plays a key role in multidecadal variability^26^. This scale signal that has a period of 67.7 years in the CET dataset is regarded as a harmonic of the solar cycle because of the harmonic relationship with the Hale sunspot cycle. Note that a quasi-millennial cycle could also be forced on the Sun by the rotation of the Trigon of the great conjunctions of Jupiter and Saturn^18^. These results clearly indicate that both solar and climate oscillations are linked to planetary motion.

Identifying causality in complex climate systems can be difficult; therefore, this study is a further attempt to better understand causality using the longest instrumental time series of temperature based on observed climate data associated with climate change. Such an approach may provide another method to study causality in climate change. As an alternative approach to GCMs, the technique directly utilizes the observed nonstationary data to directly construct the driving forces, referred to as the ‘inverse problem’ in mathematics.

It has been shown in a number of other fields^27^,^28^,^29^ that SFA can be applied to nonstationary time series to estimate a single underlying driving force with high accuracy. However, application in the climate sciences, which involves nonlinear and complex systems, is at a preliminary stage. There are uncertainties related to observational limitations, as well as missing or uncertain external forces. In particular, SFA may not account for possible nonlinear interactions between the different scales. In addition, this study used the longest instrumental record in central England, in which different sources of uncertainty may exist. Further work to evaluate this source of uncertainty is therefore desirable. These issues, among others, will be considered in forthcoming studies.

## Method

As the world’s longest instrumental record of temperature, the Met Office Hadley Centre’s CET time series represents the monthly mean surface air temperature averaged over the English midlands and spans the period January 1659 to December 2013. The record covers several episodes of natural and anthropogenic warming of multidecadal durations, and has value as one of the longest instrumental temperature records, even though it is limited in spatial extent.

SFA and wavelet analysis were used in this study. Wavelet analysis is used to detect localized structures or to analyze spectral properties. Applications of the wavelet transform technique to analyses of geophysical time series have been wide ranging. SFA is a method for extracting slowly varying driving forces from a quickly varying time series. This method has been applied to nonstationary time series with some success and can be applied to nonstationary time series to estimate a single underlying driving force with high accuracy^24^,^25^,^26^. The details of SFA can be found in literature (Wiscott, 2003)^9^.

For a given nonstationary time series 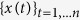, the SFA algorithm could be simply described as follows:

At first, embed the above time series into an m-dimensional space


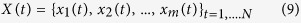


Where N = n-m + 1, n is the length of the time series and m is the embedded dimension. Second, generate an expanded space H(t) by using all monomials of orders no more than 2 generated by eq. (9), i.e.


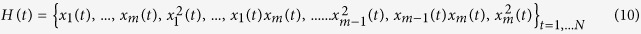


or rewrite it as


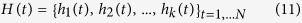


Here 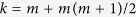. Third, normalize eq. (11) on an unit sphere then orthogonalize, denote it as


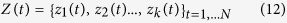


Here 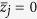, 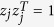.

So far, the output signals can be defined as a linear combination of the coordinate components of Z(t), which are expressed as:


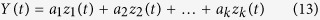


where 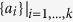 is a set of weighting coefficients.

If using 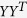 measures the varying rate of Y(t), then finding the most slowly varying signal converts to a problem of variation optimization, i.e. finding a proper 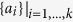 to minimize 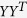. For the end, establish the covariance matrix 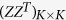, and calculate its eigenvalues 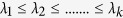 and corresponding eigenvectors. The smallest eigenvalue *λ*_1_, corresponding to the eigenvector *W*_1_, which represents the weight coefficient of the slowest varying component, then the driving forcing can be obtained by the following equation


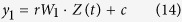


Here c and r are two given constants, and {*y*_1_(*t*)} is the output signal of the slowest driving force obtained by equation (14).

## Additional Information

**How to cite this article**: Wang, G. *et al*. Identification of the driving forces of climate change using the longest instrumental temperature record. *Sci. Rep.* **7**, 46091; doi: 10.1038/srep46091 (2017).

**Publisher's note:** Springer Nature remains neutral with regard to jurisdictional claims in published maps and institutional affiliations.
